# Overweight and obesity trends and associated factors among reproductive women in Ethiopia

**DOI:** 10.1080/16549716.2024.2362728

**Published:** 2024-06-12

**Authors:** Ermias Tadesse Beyene, Seungman Cha, Yan Jin

**Affiliations:** a Department of Human Ecology and Technology, Graduate School of Advanced Convergence, Handong Global University, Pohang, South Korea; b Department of Global Development and Entrepreneurship, Graduate School of Global Development and Entrepreneurship, Handong Global University, Pohang, South Korea; c Department of Microbiology, Dongguk University College of Medicine, Gyeongju, Korea

**Keywords:** Overweight, obesity, Ethiopia, demographic and health survey, trend

## Abstract

**Background:**

In low- and middle-income countries, the double burden of malnutrition is prevalent. Many countries in Africa are currently confronted with overweight and obesity, particularly among women, coupled with an increase in the prevalence of non-communicable diseases.

**Objective:**

This study examines trends in overweight and obesity among Ethiopian women of reproductive age from 2005 to 2016, and identifies associated factors.

**Methods:**

We used three consecutive datasets from 2005 (*n* = 14070), 2011 (*n* = 16515), and 2016 (*n* = 15683) demographic health survey years. Multilevel logistic regression was used to identify the determinant factors among individual- and cluster-level variables.

**Results:**

The prevalence of overweight and obesity among reproductive women in Ethiopia increased steadily from 6.09% in 2005 to 8.54% in 2011, and 10.16% in 2016. However, mixed patterns were observed among the regions of the country. We found that age, education, living in urban areas, and living in a rich community are associated with becoming overweight and obese. For instance, the odds of becoming overweight and obese among women aged 35–49 were higher than those among women aged 15–24 (odds ratio [OR] = 3.62, 95% Confidence Interval [CI]:2.64–4.97). Women who completed secondary school have higher odds than those without formal education (OR = 1.64, 95% CI:1.19–2.26).

**Conclusion:**

To our knowledge, this is the first study to investigate trends in the nationwide prevalence of overweight and obesity and the associated factors among Ethiopian women. This study warrants further follow-up research to identify the pathways between overweight and obesity and their probable factors.

## Paper context

- **Main findings:** The trend in the prevalence of overweight and obesity among the reproductive age women in Ethiopia showed a steady rise.
- **Added knowledge:** This study filled the research gap by analyzing the trend in the prevalence and the regional variation of overweight and obesity in the country.
- **Global health impact for policy and action:** The findings will help design appropriate policies that address the varying trends and prevalence in overweight and obesity among regions as well as the associated factors.

## Background

Obesity is a pressing global public health issue [1]. Over the past three decades, the prevalence of obesity has doubled or tripled in many countries, most likely due to sedentary lifestyles, urbanization, and the rising consumption of processed foods with high calorie content [2]. According to the World Health Organization (WHO), overweight and obesity are defined as abnormal or excessive fat accumulation that may impair health [3]. In 2016, an estimation of more than 1.9 billion adults worldwide were overweight. Among which over 650 million were obese globally [3]. In 2021, WHO reported that as a result of overweight and obesity, at least 2.8 million people are dying each year [4]. Moreover, overweight and obesity are significant risk factors for coronary heart disease, hypertension, stroke, type 2 diabetes, dyslipidemia, cancer, and other untreated non-communicable diseases (NCDs) [5–8]. Globally, approximately 120 million disability-adjusted life years (DALY) and 4.0 million deaths are attributed to overweight and obesity [9]. Although considered once as a sign of wealth and prosperity, being overweight and obese are now widely recognized as severe health problems, particularly those who are in low and lower-middle-income countries [10,11]. In addition, eight out of ten countries that contribute to more than 50% of the world’s obesity (671 million obese people) are low- and middle-income countries, namely, Brazil, Russia, India, China, Egypt, Indonesia, Mexico, and Pakistan [12]. These countries are experiencing a rapid increase in NCD risk factors, while also struggling to deal with infectious diseases and undernutrition in rural areas [13]. The ‘double burden’ of this disease is pronounced in many low-income and middle-income countries.

Similarly, many countries in Africa are currently confronted with overweight and obesity, particularly among women, coupled with an increase in the prevalence of NCDs [14,15]. In Africa, the prevalence of obesity among women is approximately double that of men [16]. This is particularly true in urban areas. The prevalence of overweight and obesity among women of reproductive age was 87% in South Africa, 74.1% in Tanzania, 66.7% in Nigeria, and 57.4% in Uganda [17]. According to a review conducted in Ethiopia, the most common risk factors for NCDs are overweight and obesity [18]. The prevalence of overweight and obesity has increased in Ethiopia [19,20]. Other studies reported that the prevalence of overweight and obesity is high among women of reproductive age in Ethiopia [21–23]. In addition, education is a substantial individual-level factor that determines the prevalence of overweight and obesity in a country [24–26]. Place of residence has also been identified as a substantial contextual factor in determining overweight and obesity [24,25]. However, these studies did not perform a trend analysis of the prevalence of overweight and obesity among women of reproductive age living in rural and urban areas [24–26]. To the best of our knowledge, studies examining the trends in overweight and obesity among women of reproductive age across Ethiopia using longitudinal datasets are scarce. Therefore, this study aimed to explore temporal trends in the prevalence of overweight and obesity and their associated factors among women of reproductive age in Ethiopia. Understanding temporal trends in the prevalence of overweight and obesity and their associated factors will have important implications for preventing and controlling emerging public health challenges in Ethiopia.

To date, five Ethiopian demographic health surveys (DHS) have been conducted, including the 2019 Ethiopia Mini DHS (EMDHS). The first survey was conducted in 2000. However, because of the unavailability of the necessary indicator (body mass index [BMI] measurement) in the original dataset of the survey years, 2000 and 2019, we could only analyze the data from the other three surveys (2005, 2011, and 2016). The primary objective of this study was to investigate the trends and factors associated with being overweight and obese among women of reproductive age in Ethiopia from 2005 to 2016. The specific objectives of this study were (1) to assess the trends in the prevalence of overweight and obesity among non-pregnant and non-postpartum women aged 15–49 years in Ethiopia from 2005 to 2016, (2) to explore the regional patterns in the time trend of the prevalence of overweight and obesity, and (3) to identify the major individual- and regional-level determinants of the prevalence of overweight and obesity.

## Methods

### Study area

Ethiopia is located in the northeastern part of Africa, known as the Horn of Africa, and lies between 30 and 150 °N latitudes and 330 and 480 °E longitudes. The country is divided into 10 regions (killils): Afar, Amhara, Benishangul-Gumuz, Gambella, Harai, Oromiya, Southern Nations, Nationalities, and People’s Region (SNNPR), Somali, Tigray, and the recently added one (Sidama), and two city administrations, Addis Ababa and Dire Dawa. However, as the Ethiopian demographic and health surveys (EDHS) dataset used in this study did not provide data for the Sidama region, we did include this region in our analysis. Ethiopia shares boundaries with Eritrea in the north, Sudan and South Sudan in the west, Kenya in the south with Kenya, and Djibouti and Somalia in the east.

### Ethiopian demographic and health surveys 2005–2016

We used data from the EDHS for three consecutive survey years (2005, 2011, and 2016). The EDHS is a population-based cross-sectional survey conducted across Ethiopia. The sample selection was conducted using a two-stage stratified cluster sampling procedure. In the first stage, enumeration areas (EAs), which are geographic areas consisting of convenient numbers of dwelling units serving as counting units for the census, were selected from the nine regions and Dire Dawa city administration except Addis Ababa (entirely urban). An EA is a geographic area of 200–300 households that serves as a counting unit for the census [27]. The EAs were chosen from a list authorized by the Ethiopian Central Statistics Agency as a sampling framework for the Population and Household Census (PHC). The second stage involved selecting a specified number of households from each EA or cluster using a systematic sampling technique. Details of the sampling design can be found in the EDHS reports for successive survey years [28–30].

**Figure 1.**
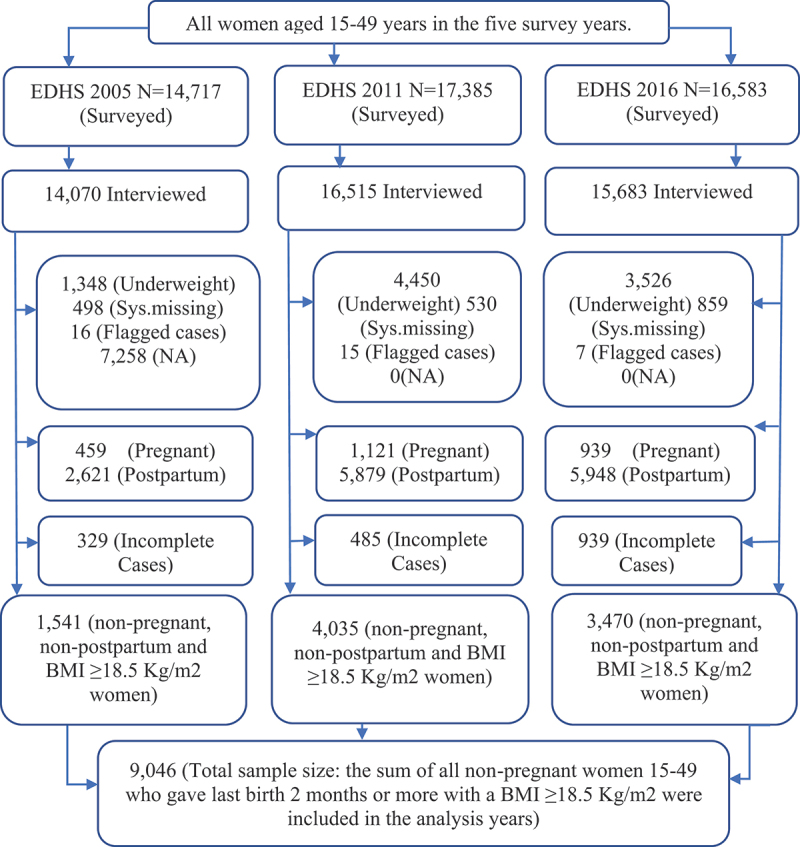
Flow chart for the selection of study participants.

The three EDHSs surveyed 14,070, 16515, and 15,683 women of reproductive age (15–49 years) in 2005, 2011, and 2016, respectively. All women 15–49 years old who were eligible for the surveys were selected for the woman’s questionnaire, which collected data on BMI and other issues. The surveys were conducted from 27 April to 30 August 2005; 27 December 2010, to 3 June 2011; and 18 January 2016, to 27 June 2016. To extract the working dataset, we first eliminated underweight women and those with missing, flagged, or unavailable data. Next, to prevent potential weight gain related to pregnancy, we excluded non-pregnant and non-postpartum women. As a result, 459, 1,121, and 939 pregnant women were removed from the 2005, 2011, and 2016 surveys, respectively, and 2,621,5,879, and 5,948 postpartum women were removed from the 2005, 2011, 2016, and 2015 surveys, respectively. Finally, incomplete data were excluded from each survey year. The screened subtotal sample sizes for each survey year were 1,541 in 2005, 4,035 in 2011, and 3,470 in 2016. Consequently, we considered a total sample size of 9,046 non-pregnant and non-postpartum women. Figure 1 shows this information.

## Statistical analysis

### Factors of analysis

#### Dependent variable

The primary focus of our study was the prevalence of overweight and obesity among women of reproductive age, excluding those who were pregnant or postpartum. This measure was derived from the numerical variable ‘body mass index for respondent’ based on WHO standards: 1) underweight (BMI: less than 18.50), 2) normal (BMI: 18.50–24.99), 3) overweight (25.00–29.99) and 4) obese (BMI: greater than 30.00) [31]. Subsequently, we aggregated these variables to create another categorical variable with two categories (normal, overweight, and obese).

#### Independent variables

Using the EDHS, the choice of independent variables was based on previous studies and their presence in the dataset. These variables are generally classified as individual- and regional-level variables. Some variables are transformed, recategorized and coded again to fit into the multilevel analysis.

#### The individual level

At the individual level, we categorized women based on various factors: age of women (generated from the variable ‘current age of respondent’) is recategorized as 15–25, 25–34, and 35–49, women’s education level (no education, primary education, secondary education, and higher education), women’s religion (Orthodox, Catholic, Protestant, Muslim, Traditional and other), contraceptive use (no method, folkloric method, traditional, and modern method), marital status (recategorized as: in a union, not in a union), women work status (has work, has no work), household wealth index (recategorized as: poorest or poorer, middle, and richer or richest), media access (listening radio, reading newspaper/magazine, and watching television), parity (the number of children a woman has) and partners education.

#### The regional-level

We used the two variables region, place of residence and survey years directly from the dataset, but transform and aggregate, recategorize and recode the individual-level variables (education level and wealth status) to obtain the other two regional-level variables, namely, community women education level, and community women’s wealth status.

To obtain the regional-level educational status, we combined the individual-level educational status by calculating the percentage of non-pregnant and non-postpartum women who had completed at least primary school at the regional level and classified this variable as high (>50%) or low (≤50%). Similarly, we first aggregate the individual wealth index categorical variables to obtain the regional wealth index by calculating the mean wealth index at the regional level. We classified communities with a wealth index below the mean value as poor. Similarly, a community with a wealth index above the mean value, is classified as rich. The variable region represents nine administrative regions: Tigray, Afar, Amhara, Oromia, Somali, Benishangul-Gumuz, the South National Nationalities People’s Region (SNNPR), and two city administrations (Addis Ababa and Dire Dawa). The place of residence indicated whether the respondent lived in an urban or rural area. It determines a person’s access to services, information about health, and other aspects of life [30].

#### Data analysis

To analyze the trends in the prevalence of overweight and obesity and the associated factors in each survey year, our outcome of interest (indicator) was the prevalence of overweight and obesity. The DHS provided a weighting factor. Weighting was used because of possible disparities in response rates and the non-proportional allocation of the sample to different regions, including urban and rural areas. A complex survey sampling technique was used to analyze the weighted data. We conducted significance tests and associations between the response and explanatory variables based on these assumptions. To quantify the significant associations, OR with 95% confidence interval [CI] was used.

#### The time trend regional variation in prevalence of overweight and obesity

To investigate regional variations in time trends, we used descriptive statistics that showed the percentages of overweight and obesity prevalence in each region in a specific year. To elaborate on the regional variation and the trend during the study period, we calculated the percentage point (pp) change in 2005−2011, 2011−2016, and 2005–2016. Moreover, we display regional variations and trends in the graphs.

#### Factors associated with prevalence of overweight and obesity

To identify the major factors determining the prevalence of overweight and obesity, we employed a multi-level mixed effect logistic regression analysis using the R software version (4.1.2 (1 November 2021)). In the process of our analysis to assess the association between the dependent and independent variables of the study we used two-level bivariate mixed-effect logistic regression. In the final multi-level mixed effect logistic regression model, we included categorical variables with a *p* value of < 0.25 and used the estimated log odds ratio with a 95% CI to declare whether the variable was an independent variable. Statistical significance was set at *p* < 0.05. To select the best-fitting model, we constructed four consecutive models and evaluated their Akaike Information Criterion (AIC) and Bayesian information criterion (BIC) values. The first model was the intercept only (null/empty model), and no explanatory or exposure variables were included to show the total variance between communities. The second was a fixed effects model encompassing all individual-level variables that were initially significant, with a *p* value of < 0.25. The third model is a random-effects model, which has only regional-level variables. The final/fourth model is a mixed-effect model with individual-level variables (fixed effect) and regional-level variables (random effect). The model used for the analysis is as follows:

$$\mathrm{Logit}\;\left(P_{\mathrm{ij}}\right)\;=B_{00}+B_{10}*x_{\mathrm{ij}}+B_{01}*X_{j}+U_{0j}+e_{\mathrm{ij}}$$

Where i and j are the individual (Level 1) and community (Level 2) units, respectively. P_ij_ is the probability of the outcome of interest for woman i in community j; *B*s are the fixed coefficients (AOR); x and X represent the individual- and regional-level independent variables, respectively; *U* indicates the random effects for the j^th^ community; and ε shows the unmeasured factors that may influence the primary outcome of interest.

Descriptive statistics were used to analyze the means, standard deviations, proportions, and percentages of the study variables. To assess individual variation, we analyzed the fixed effect using an adjusted odds ratio along with 95% confidence intervals (CI), whereas for regional-level (random effect), we computed the intraclass correlation coefficient (ICC) value to examine the clustering effect and the degree to which the unexplained variance of the empty model is explained by the regional-level factors using the following statistical formula:

$$\mathrm{ICC}=\frac{\delta^{2}a}{\delta^{2}a+\delta^{2}b}$$

where $\delta^{2}a$ and $\delta^{2}b$ indicate the regional- and individual-level variances, respectively. Individual-level variance $\delta^{2}b$ is a fixed value of $\frac{\pi^{2}}{3}$.

The goodness of fit of the adjusted final model was estimated using the AIC and BIC statistics and checked using a log-likelihood test compared to the preceding models. The smaller the values of AIC and BIC, the better the model fit. In addition, any interaction among the predicator variables was tested, and there was no significant interaction between the individual- and regional-level variables.

## Results

### Background characteristics of the study participants

The background characteristics of the participants are listed in Table 1. Women aged 25–34 were the highest in proportion, accounting for 40.4%, 42.2%, and 46.7% in the three successive survey years, respectively. The proportion of women with no formal education decreased from 74.63% in 2005 to 62.93% in 2016. The proportion of those with primary and higher education rose from 17.08% in 2005 to 27.54% in 2016, and from 1.11% in 2005 to 3.24% in 2016. The proportion of adult men without any formal education decreased from 58.81% in 2005 to 47.14% in 2016, whereas those with higher education rose from 2.71% in 2000 to 6.41% in 2016. Concerning religion distribution/composition, followers of Orthodox Christianity decreased from 49.38% in 2005 to 44.12% in 2016.

**Table 1.** Background characteristics (weighted %) of non-pregnant and non-postpartum women of the Ethiopian demographic and health survey years 2005, 2011, and 2016 (N = 9046).

Variables	Survey year						Total	
	2005 (*n* = 1541)		2011 (*n* = 4035)		2016 (*n* = 3470)		2005–2016 (*N* = 9046)	
	Freq.	%	Freq.	%	Freq.	%	Freq.	%
Age category								
15–24	284	19.80	732	18.09	537	15.66	1553	17.85
25–34	634	40.40	1694	42.23	1584	46.66	3912	43.10
35–49	623	39.80	1609	39.69	1349	37.68	3581	39.06
Educational status								
No education	1098	74.63	2518	66.18	2055	62.93	5671	67.91
Primary	250	17.08	1085	26.90	954	27.54	2289	23.84
Secondary	162	7.19	258	3.79	292	6.29	712	5.76
Higher	31	1.11	174	3.13	169	3.24	374	2.49
Religion								
Orthodox	774	49.38	1829	46.38	1411	44.12	4014	46.63
Catholic	16	1.24	40	0.97	20	0.73	76	0.98
Protestant	245	17.80	721	23.72	661	24.01	1627	21.84
Muslim	469	29.01	1378	26.96	1332	29.60	3179	28.52
Other	37	2.56	67	1.97	46	1.54	150	2.02
Contraceptive use								
No method	1187	79.24	2631	63.87		56.97	3818	66.69
Folkloric method			1	0.03	2012	0.62	2013	0.22
Traditional method	26	0.90	66	1.13	33	42.41	125	14.81
Modern method	328	19.86	1337	34.98	1425		3090	18.28
Marital status								
In a Union	1305	85.34	3398	85.80	3470	87.81	8173	86.32
Not in a Union	236	14.01	637	14.20		11.28	873	13.16
Women work status								
No	1085	69.32	2461	60.24	2241	64.26	5787	64.61
Yes	456	30.68	1574	39.76	1229	35.74	3259	35.39
Wealth status								
Poorest or poorer	595	38.81	1476	38.27		37.35	2071	38.14
Middle	195	13.40	598	19.43	3470	19.59	4263	17.47
Richer or richest	751	47.79	1961	42.29		43.05	2712	44.38
Read newspaper/magazine								
No	1511	99.04	3878	97.27	3378	97.97	8767	98.09
Yes	30	0.96	157	2.73	92	2.03	279	1.91
Listen to radio								
No	1279	85.83	3168	79.98	2938	85.71	7385	83.84
Yes	262	14.17	867	20.02	532	14.29	1661	16.16
Listen/watch TV								
No	1394	94.94	3203	86.81	2764	87.24	7361	89.66
Yes	147	5.06	832	13.19	706	12.76	1685	10.34
Parity								
No child	26	2.10	51	0.95	25	0.71	102	1.25
1 to 3 children	791	48.05	2214	51.88	1757	50.73	4762	50.22
4 and above children	724	49.86	1770	47.17	1688	48.57	4182	48.53
Partner’s education								
No education	876	58.81	1857	48.38	1574	47.14	4307	51.44
Primary	362	26.10	1447	39.12	1147	37.26	2956	34.16
Secondary	243	12.38	423	6.60	416	9.19	1082	9.39
Higher	60	2.71	308	5.90	333	6.41	701	5.01
Region								
Tigray	133	5.12	442	6.05	336	6.53	911	5.90
Afar	80	1.03	239	0.65	205	0.63	524	0.77
Amhara	299	28.14	635	27.87	502	26.39	1436	27.47
Oromia	301	35.54	605	36.39	491	34.80	1397	35.58
Somali	98	3.59	241	1.81	331	2.57	670	2.66
Ben-Gumz	89	0.88	355	1.04	297	0.97	741	0.96
SNNPR	288	20.91	633	21.29	563	22.56	1484	21.59
Gambela	28	0.30	137	0.26	112	0.27	277	0.28
Harari	20	0.26	174	0.17	102	0.22	296	0.22
Addis Ababa	162	3.74	370	4.19	307	4.53	839	4.15
Dire Dawa	43	0.49	204	0.29	224	0.54	471	0.44
Place of residence								
Urban	370	14.42	1268	20.86	995	19.27	2633	18.18
Rural	1171	85.58	2767	79.14	2475	80.73	6413	81.82
Community-level education								
Low	1106	78.30	2257	60.67	1847	57.23	5210	65.40
High	435	21.70	1778	39.33	1623	42.77	3836	34.60
Community-level wealth								
Low	758	54.09	2123	63.21	1843	58.1	4724	58.45
High	783	45.91	1912	36.79	1627	42	4322	41.55

### The time trend in the prevalence of overweight and obesity per region and by women characteristics

**Figure 2.**
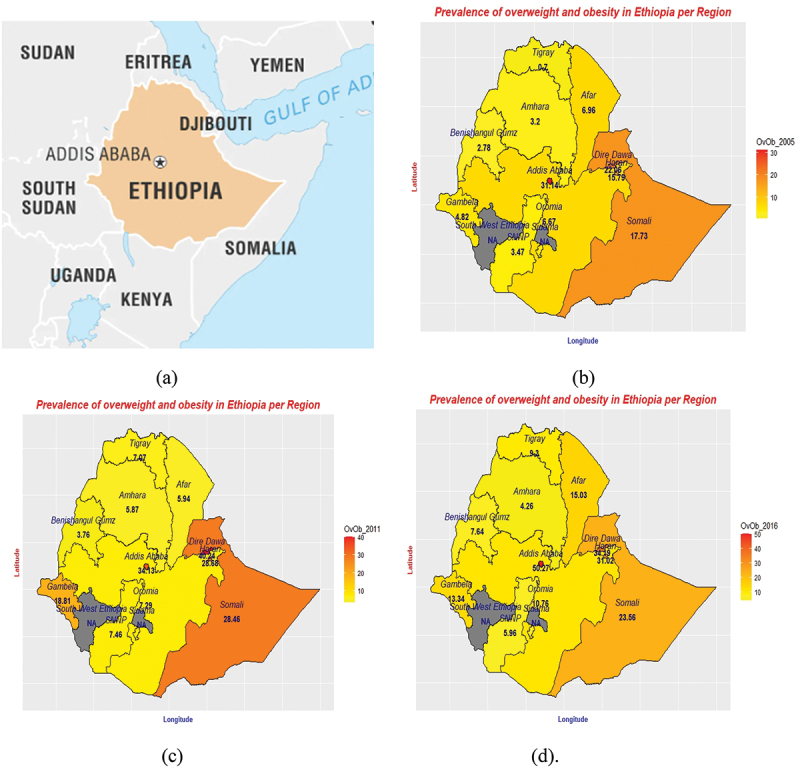
Location of Ethiopia (a) and Prevalence of overweight and obesity by region 2005(b), 2011(c), 2016(d) (Figure 2(a). A source: Britannica). OvOb: Overweight and Obesity.

Table 2 and Figures 2 show that the proportion of overweight and obese women has increased over the past decade (2005–2016). At the national level, the prevalence of overweight and obesity increased steadily from 6.09% in 2005 to 8.54% in 2011, and 10.16% in 2016. Looking at the time trend in 2005–2011, all regions except Afar showed an increase in the prevalence of overweight and obesity. The Dire Dawa, Gambela, and Harari regions showed the highest percentage point (pp) changes in this period, accounting for 18.18pp, 13.99pp, and 12.89pp, respectively. However, the Afar region showed the lowest pp change (1.02pp). In 2011–2016, some regions and the Dire Dawa City administration showed a decline in the prevalence of overweight and obesity. The greatest decline in the prevalence of overweight and obesity was observed in Dire Dawa (6.05pp), followed by Gambela (5.47pp) and Somali (4.90pp). However, in the same period, some regions and the Addis Ababa City administration showed an increase. The highest pp change was observed in Addis Ababa, Afar, and Benishagul-Gumuz, accounting for 16.14pp, 9.09pp, and 3.88pp change, respectively. From 2005 to 2016, all regions showed an increase in the prevalence of overweightness and obesity. The Addis Ababa, Harari, and Dire Dawa city administrations registered the highest prevalence changes, accounting for 19.13pp, 15.23pp, and 12.13pp, respectively. The smallest change was observed in Amhara. More details on the trend in the prevalence of overweight and obesity are found in the Supplementary Material (Text S1 and Figure S1).

**Table 2.** Trends in the prevalence of overweight and obesity among non-pregnant women aged 15–49 per region (weighted) in the years 2005, 2011, and 2016 (*N* = 9,046).

Characteristics	2005(%)	2011(%)	2016(%)	2011–2005 pp	2016–2011 pp	2016–2005 pp 95%CI
Region						
Tigray	0.70	7.07	9.30	6.37	2.23	8.60 (8.31, 8.89)
Afar	6.96	5.94	15.03	−1.02	9.09	8.07 (7.49, 8.65)
Amhara	3.20	5.87	4.26	2.67	−1.61	1.06 (0.88, 1.24)
Oromia	6.67	7.29	10.76	0.62	3.47	4.09 (3.63, 4.55)
Somali	17.73	28.46	23.56	10.73	−4.90	5.83 (4.68, 6.98)
Ben-Gumz	2.78	3.76	7.64	0.98	3.88	4.86 (4.60, 5.12)
SNNPR	3.47	7.46	5.96	3.99	−1.50	2.49 (2.26, 2.72)
Gambela	4.82	18.81	13.34	13.99	−5.47	8.52 (8.04, 9.00)
Harari	15.79	28.68	31.02	12.89	2.34	15.23 (13.96, 16.50)
Addis Ababa	31.14	34.13	50.27	2.99	16.14	19.13 (16.88, 21.38)
Dire Dawa	22.06	40.24	34.19	18.18	−6.05	12.13 (10.58, 13.68)
Ethiopia	6.09	8.54	10.16	2.45	1.62	4.07 (3.65, 4.49)

**SNNPR, Southern Nations, Nationalities, and Peoples’ Region**.

Overall, between 2005 and 2016, Addis Ababa, Harari, Dire Dawa, Tigray, Benishagul-Gumuz, and Oromia showed a steady increase in the prevalence of overweight and obesity among women of reproductive age. The remaining exhibited mixed patterns (rise and fall) during this period. Time trends in the prevalence of overweight and obesity per region are shown in Table 2 and for better visualization is graphically illustrated using maps in Figure 2.

Moreover, the time trend in the prevalence of overweight and obesity varied according to the characteristics of women across the survey years. There was an increase in the prevalence of overweight and obese individuals across age groups. The highest increase was observed among women aged 25–34 (6.55%p) who had completed primary education (5.33%p), were Catholic believers (7.81%p), had worked (7.19%), and were rich (13.17pp). Besides looking at the effect of media access, women who read newspapers/magazines, listened to radio, and watched TV had shown an increase in the prevalence of overweight and obesity between 2005 and 2016 by 14.95pp, 9.53pp, and 3.80%p, respectively. Furthermore, the trend showed that the prevalence of obesity increased from 23.55% in 2005 to 32.56% in 2016 with an overall 9.01pp (95% CI 7.44–10.58) in urban areas, whereas it increased from 3.15% in 2005 to 4.81% in 2016 with an overall 1.66pp (95% CI 1.47–1.85) in rural areas. More details of the trend analysis by each female characteristic are described in the Supplementary Material (Table S1).

### Individual- and regional-level factors influencing the prevalence of overweight and obesity among non-pregnant and non-postpartum women of reproductive age

#### Model assessment

The ICC value in the null model of the pooled dataset indicated that 19.80 of the total variances in the odds of becoming overweight or obese was due to between-community characteristics. The remaining 80.20% of the total variability in the prevalence of overweight and obesity is attributed to individual-level differences. The cluster variability/ICC declined over successive models from 19.80% to 9.35%, to approximately 0. Compared to the preceding models, Model 4 (the combined model), which consisted of individual- and regional-level factors, showed the smallest AIC and BIC values. Therefore, we adopted a combined model for our analysis to predict overweightness and obesity in women.

#### Factors associated with overweight and obesity

The multilevel analysis showed that all the individual-level variables: age, education level, religion, read newspaper/magazine, listen/watch TV, parity, and education of partner were found to be significant determinant factors of overweight and obesity among the non-pregnant and non-postpartum women. Between 2005 and 2016 women aged 25–34 and 35–49 were more likely to be overweight and obese, with odds ratio of 2.38 (95% CI: 1.79–3.18) and 3.62 (95% CI: 2.64–4.97), respectively, than those aged 15–24. In the same period, women who attended secondary school had higher odds of being overweight and obese compared to those who had no education (OR = 1.64; 95% CI:1.19–2.26).

Result showed that women who followed traditional beliefs were more likely to be overweight and obese with an OR = 3.95 (95% CI: 2.15–7.25) compared to those who were Orthodox believers. Women who had one to three children were significantly less likely to be overweight and obese than those who had no child with an OR = 0.32 (95% CI: 0.17–0.61). Those with at least four children were 66% less likely to be overweight or obese (OR = 0.34; 95% CI: 0.18–0.65). The other individual-level variable that was found to be significant is women partners’ education level, hence women whose partner finished high school were 37% (OR = 1.37; 95% CI: 1.03–1.84) more likely to be overweight and obese than those women whose partner had no formal education. While those women whose partner has completed higher education were 2.09 times (95% CI: 1.50–2.92) more likely to be overweight and obese than those women whose partner had no formal education.

Moreover, the regional-level variables of region, place of residence, education, and wealth were significantly associated with the prevalence of overweight and obesity in the survey years. The odds of the results are presented in Table 3. Women who lived in the Oromia, Somali, Benishagul-Gumuz, Gambela, and Harari regions and the Addis Ababa and Dire Dawa city administration were more likely to be overweight and obese than those who lived in the reference region (Tigray). Those who lived in the Somali region were eight times (95% CI: 4.80–13.34) more likely to be overweight or obese than those who lived in Tigray. Moreover, those women who lived in Harari were 3.10 times (95% CI: 0.90–10.67) more likely to be overweight and obese than those women who lived in Tigray. However, women who lived in Benishagul-Gumuz were significantly less likely to be overweight and obese than those who lived in Tigray by 79% (OR = 0.21; 95% CI: 0.03–1.24). In addition, women who lived in the urban area were significantly more likely to be overweight and obese (OR = 2.80; 95% CI: 2.15–3.66) than those women who lived in the rural area.

**Table 3.** Multilevel mixed effect logistic regression analysis of individual- and regional-level factors associated with prevalence of overweight and obesity among reproductive age women in Ethiopia, 2005, 2011, and 2016.

Characteristics fixed effect	Null Model	Model I AOR (95% CI)		Model II AOR (95%CI)		Model III AOR (95%CI)	
**Age category**							
15-24 (ref)		1				1	
25-34		2.91	(2.16, 3.93)***			2.38	(1.79, 3.18)***
35-49		4.59	(3.30, 6.40)***			3.62	(2.64, 4.97)***
**Educational status**							
No education (ref)		1				1	
Primary		1.40	(1.13, 1.74)**			1.11	(0.89, 1.38)
Secondary		2.25	(1.61, 3.15)***			1.64	(1.19, 2.26)**
Higher		1.41	(0.91, 2.18)			1.04	(0.69, 1.56)
**Religion**							
Orthodox (ref)		1				1	
Catholic		0.46	(0.17, 1.23)			0.46	(0.18, 1.17)
Protestant		0.80	(0.63, 1.03)			0.79	(0.62, 1.01)
Muslim		0.83	(0.66, 1.04)			0.83	(0.67, 1.04)
Traditional & other		1.72	(1.00, 2.95)*			3.95	(2.15, 7.25)***
**Contraceptive use**							
No method (ref)		1				1	
Traditional method		0.61	(0.31, 1.21)			0.68	(0.38, 1.21)
Modern method		1.08	(0.90, 1.29)			0.95	(0.80, 1.13)
**Marital status**							
In a Union (ref)		1				1	
Not in a Union		0.79	(0.58, 1.07)			0.80	(0.59, 1.07)
**Women work status**							
No (ref)		1				1	
Yes		1.02	(0.86, 1.21)			0.93	(0.79, 1.10)
**Wealth status**							
Poorest or poorer (ref)		1				1	
Middle		0.63	(0.44, 0.90)**			0.84	(0.57, 1.23)
Richer or richest		1.31	(1.10, 1.57)**			1.08	(0.81, 1.45)
**Read newspaper/magazine**							
No (ref)		1				1	
Yes		1.96	(1.35, 2.86)***			1.92	(1.36, 2.71)***
**Listen radio**							
No (ref)		1				1	
Yes		1.06	(0.87, 1.30)			1.07	(0.88, 1.29)
**Listen/watch TV**							
No (ref)		1				1	
Yes		3.17	(2.54, 3.97)***			1.79	(1.44, 2.23)***
**Parity**							
No child (ref)		1				1	
1 to 3 children		0	(0.18, 0.64)***			0.32	(0.17, 0.61)***
4 and above children		0.31	(0.16, 0.59)***			0.34	(0.18, 0.65)**
**Partner’s education**							
No education (ref)		1				1	
Primary		1.22	(0.98, 1.51)			1.04	(0.84, 1.29)
Secondary		1.90	(1.40, 2.58)***			1.37	(1.03, 1.84)*
Higher		3.07	(2.16, 4.36)***			2.09	(1.50, 2.92)***
**Region**							
Tigray (ref)				1		1	
Affar				245.82	(108.94, 554.67)***	0.90	(0.28, 2.89)
Amhara				0.96	(0.66, 1.39)	1.04	(0.71, 1.53)
Oromia				1.68	(1.18, 2.39)**	1.79	(1.23, 2.61)**
Somali				6.40	(4.02, 10.20)***	8.00	(4.80, 13.34)***
Ben-Gumz						0.21	(0.03, 1.24)
SNNPR				1.18	(0.81, 1.72)	1.34	(0.88, 2.03)
Gambela				190.95	(31.86, 1144.38)***	2.96	(0.84, 10.44)
Harari						3.10	(0.90, 10.67)
Addis Ababa				2.73	(1.86, 4.01)***	2.45	(1.63, 3.68)***
Dire Dawa				0.01	(0.00, 10.40)	2.86	(1.22, 6.68)*
**Place of residence**							
Urban (ref)				1		1	
Rural				0.22	(0.18, 0.28)***	0.36	(0.28, 0.46)***
**Regional-level education**							
Low (ref)				1		1	
High				1.61	(1.29, 2.02)***	1.41	(1.10, 1.80)**
**Regional-level wealth**							
Low (ref)				1		1	
High				1.74	(1.43, 2.12)***	1.61	(1.28, 2.02)***
**Survey Year**							
2005 (ref)				1		1	
2011				1.05	(0.83, 1.32)	1.13	(0.88, 1.45)
2016				1.30	(1.03, 1.64)*	1.33	(0.97, 1.82)
AIC	5210.17		4541.37		4554.35		4361.44
BIC	5224.39		4740.45		4675.22		4667.17
Log Likelihood	-2603.08		-2242.68		-2260.18		-2137.72
Num. obs.	9046		9046		9046		9046
Num. groups: V024	11		11		11		11
Var: V024 (Intercept)	1		0.34		0		0

AOR, Adjusted odds ratio; CI, confidence interval; SNNPR, Southern Nations, Nationalities, and Peoples’ Region. Significant codes: ‘***’ p<0.001, ‘**’ p<0.01, ‘*’ p<0.05, ref=reference category.

Furthermore, women who belonged to a community with high level of education were significantly more likely to be obese by 59% (OR = 1.41; 95% CI: 1.10–1.80) than those women who belonged to less educated community. Considering the regional-level wealth status, women who belong to the rich community were 1.61 times (95% CI: 1.28–2.02) more likely to be overweight and obese than their counterparts.

## Discussion

The time trend analysis of the prevalence of overweight and obesity in Ethiopia showed a steady rise in consecutive survey years (2005–2016) by a significant percentage point. This is consistent with findings from neighboring countries, current trends in most low-income and middle-income countries, and across the globe. However, mixed patterns (rise and fall) were observed among the regions of the country.

In addition, looking at the time trends for each period (2005–2010 and 2011–2016), the patterns varied by region. For instance, Addis Ababa, Harari, Dire Dawa, Tigray, Benishagul-Gumuz, and Oromia showed a steady increase in the prevalence of overweight and obesity among women of reproductive age in both periods, whereas the rest showed a mixed pattern with fluctuations. In 2005–2011, the Dire Dawa, Gambela, and Harari regions had the greatest percentage point (pp) changes during these periods, whereas the Afar region showed the lowest pp change. In 2011–2016, the Addis Ababa, Harari, and Dire Dawa showed the greatest change in prevalence, whereas the least change was observed in the Amhara region. A possible reason for this is that both Addis Ababa and Dire Dawa, including Harar, are major metropolitan cities in Ethiopia with large populations. Moreover, urbanization was faster in these three areas than that in other areas.

The multilevel analysis revealed that the individual-level factors (age, women’s education level, religion, read newspaper/magazine, listen/watch TV, parity, and partners education) were associated with overweight and obesity among women in Ethiopia. Women aged 25–34 years and 35–49 years were more likely to be overweight and obese than those aged 15–24 years. This result is consistent with those of previous studies [24,32]. A possible reason for this may be the high rates of physical inactivity and increased consumption of high-energy foods associated with aging, which can lead to overweight and obesity [25]. This can also be explained by a shift in body composition caused by age-related hormonal changes and/or an increase in and redistribution of body fat [33,34].

Women who had completed secondary school were more likely to be overweight or obese than those without education. This finding is consistent with many previous studies [35–48]. However, some countries have found the strongest evidence of a negative correlation between these two factors. For example, a study conducted in China found an inverse association between educational level and being overweight or obese [49]. This may be due to higher education and improved socio-economic status contributing to better health through increased health information and improved habits in more affluent countries [45]. In LMICs, higher educational attainment is correlated with higher socioeconomic status and access to material resources, which makes women more likely to adopt ‘Western’ diets or processed foods high in fat and refined carbohydrates and sedentary lifestyles, which increases their risk of gaining weight.

Additionally, our study revealed that women who spent more time reading newspapers and/or magazines were more likely to be overweight and obese than their counterparts. This could be because of the associated lifestyle changes while reading; that is, they become physically less active. Similarly, TV exposure had a correlation with being overweight and obese. The increased TV viewing time of women is associated with a higher likelihood of obesity due to a sedentary lifestyle. These findings are in line with other studies [26,39,40,44,45,50,51]. Advertising also increases calorie intake and can lead viewers to choose an unhealthy diet [52]. Women with at least one child were less likely to be overweight or obese than those without children. This may be due to several factors, including workload, care, production, and reproduction. However, different results were obtained in a study conducted in Nepal [38]. According to the available data, the relationship between parity and obesity differs between cultures [18], ethnic groups [15,21–23], and national development levels. This may also highlight the need for further studies in this area.

Furthermore, the multilevel analysis results showed that region was one of the determining regional-level factors for the prevalence of overweight and obesity. This result is consistent with that of a previous study conducted in Kenya [35]. We found that women who lived in the Oromia, Somali, Benishagul-Gumuz, Gambela, and Harari regions and the Addis Ababa and Dire Dawa city administration were more likely to be overweight and obese than those who lived in the reference region (Tigray). Living in urban was found to be a factor of overweight and obesity. Similar results have been reported previously [35–40,53–55]. A possible reason for the association between living in urban areas and overweight and obesity could be that urban areas experience fast-paced economic growth, in which population density rises and food consumption follows Western diets, which has become a trend in many developing countries [56–62]. The continual influx of people from rural to urban areas, resulting in high population density and high demand for housing and infrastructure, may have contributed to the drying up of open areas, which may have reduced the opportunity for residents’ physical activities [53,62]. This largely affects the lifestyle of the residents shifting outdoor activities to more home-based entertainment, leading to increased sitting time and less energy-intensive activities in the urban areas of developing countries. Unlike urban women, rural women may be less likely to gain weight because of their engagement in labor-intensive activities such as agriculture.

The study is constrained by the absence of information on variables, such as comorbidity conditions, dietary intake, physical activities, and energy expenditures in the DHS dataset, which warrants further study.

## Conclusion

The country is witnessing a health transition due to urbanization and changes in feeding habits and lifestyles, marking a significant change in the percentages of overweight and obese people. The findings of this study underscore the importance of targeted interventions and policies aimed at promoting healthier lifestyles and addressing the underlying factors driving excessive caloric consumption, varying trends, and risk factors across different regions and demographic groups. Initiatives focused on enhancing physical activity habits, improving access to nutritious foods, and increasing awareness of the risks associated with being overweight and obese could help mitigate the growing burden of noncommunicable diseases in Ethiopia. Further research is needed to explore additional factors, such as comorbidities, dietary intake, and physical activity levels to better inform comprehensive intervention strategies.

## Supplementary Material

Supplemental Material
