# Supplementary material for: Overweight and obesity trends and associated factors among reproductive women in Ethiopia
Source: Glob Health Action. 2024 Jun 12;17(1):2362728. doi: 10.1080/16549716.2024.2362728 (PMC11172244; doi:10.1080/16549716.2024.2362728)
Supplement: Supplemental Material [file ZGHA_A_2362728_SM4893.docx]

***Supplementary Text S1***

**Trends in the prevalence of overweight and obesity**

The data showed that the proportion of overweight and obese women increased over the past decade (2005, −2016). The prevalence of overweight and obesity increased steadily from 6.09% in 2005 to 8.54% in 2011 and 10.16% in 2016 (Figure S1). All regions showed an increase in the percentage of reproductive-aged women who were overweight or obese during the three survey years. Three regions–Tigray, Oromia, and Benishangul-Gumz–and two city administrations–Harari and Addis Ababa–continuously increased between 2005 and 2016. The other regions and city administrations showed a rise and fall in the prevalence of overweight and obesity. The greater proportion of women who were overweight and obese in each survey year was mostly concentrated in three administrations: Addis Ababa, Dire Dawa, and Harari. The lowest proportion of overweight and obese women during this period was observed in the Amhara region. The lowest percentage-point change in the prevalence of overweight and obesity between 2005 and 2016 was observed in Amhara.

In addition, the trend showed a significant disparity between rural and urban areas regarding the percentage change in the prevalence of overweight and obesity. The prevalence of obesity increased from 23.55% in 2005 to 32.56% in 2016 with an overall 9.01pp (95% CI: 7.44−10.58) in urban areas, while it increased from 3.15% in 2005 to 4.81% in 2016 with an overall 1.66pp (95% CI: 1.47−1.85) in rural areas. Ethiopia has shown a continual increase in the prevalence of overweight and obesity since 2000 by 4.07 percentage points(%p) (Figure S1).

***Supplementary Figure S1***


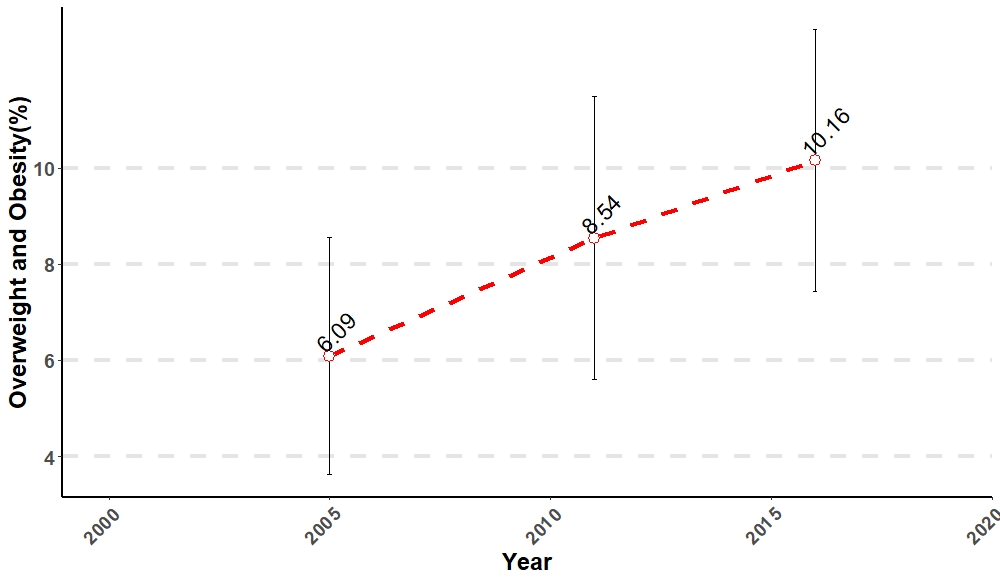


Figure S1. Trends in the prevalence of Overweight and obesity among non-pregnant and non-postpartum women aged 15-49 years from 2005-2016, Ethiopian Demographic and Health Surveys (EDHS) 2005,2011, and 2016 (N = 9,046).

***Supplementary Text S2***

Trends in the prevalence of overweight and obesity varied according to the women’s characteristics across the survey years. There was an increase in the prevalence of overweight and obesity across the age groups. The highest increment was observed among women aged 25−34 at an overall 6.55pp increment, followed by those aged 35−49 years at 2.28pp. The largest increase in the prevalence of overweight and obesity in relation to women’s education level was observed in those who had completed primary education (5.33%), followed by those with secondary education (3.70%). The number of women who completed higher education declined between 2005 and 2016 by 12.92pp. In relation to religion, the largest increase in the prevalence of overweight and obesity was registered among Catholic women, followed by Orthodox and Protestant believers at 7.81pp,5.56pp, and 3.67%, respectively. The prevalence of overweight and obesity was found to be higher in women who had worked than in those who had no work, and between 2005 and 2016, it increased by 7.19pp. While women in the poor or middle-wealth category showed a reduction in the prevalence of overweight and obesity, those who were rich showed an overall 13.17pp. Women who read newspaper/magazine, listen radio, and watch TV had shown increment in the prevalence of overweight and obesity between 2005 and 2016 by 14.95pp, 9.53pp, and 3.80%p, respectively. With respect to region, the largest increases in the prevalence of overweight and obesity were observed in the three city administrations of Addis Ababa, Harari, and Dire Dawa, by 19.13pp, 15.23pp, and 12.13%, respectively. The smallest increase was observed in the Amhara region (1.06 %) between 2005 and 2016. In addition, with regard to place of residence, a larger change in the prevalence of overweight and obesity was observed in the urban area (overall 9.00 %) compared to 1.66% in the rural area.

***Supplementary Table S1***

Table S1. Trends in the prevalence of overweight and obesity among women of reproductive age by characteristics (weighted) in 2005, 2011, and 2016

| **Characteristics** | **EDHS 2005 n(%)** | **EDHS 2011 n(%)** | **EDHS 2016 n(%)** | **Percentage point (%p) difference (2005-2016) 95%CI** | |
| --- | --- | --- | --- | --- | --- |
| **Age category** |  |  |  |  |  |
| 15-25 | 15(3.92) | 31(3.63) | 37(4.92) | 1.00 | (0.77, 1.22) |
| 25-34 | 41(5.18) | 176(8.81) | 260(11.73) | 6.55 | (6.12, 7.00) |
| 35-49 | 63(8.10) | 197(10.50) | 186(10.38) | 2.28 | (1.78, 2.78) |
| **Educational status** |  |  |  |  |  |
| No education | 55(3.81) | 168(5.38) | 174(5.82) | 2.01 | (1.77, 2.25) |
| Primary | 20(5.93) | 131(10.28) | 147(11.26) | 5.33 | (4.89, 5.78) |
| Secondary | 38(27.34) | 65(36.26) | 93(31.04) | 3.70 | (2.02, 5.38) |
| Higher | 5(24.75) | 40(26.83) | 1847(11.83) | -12.92 | (-14.19, -11.65) |
| **Religion** |  |  |  |  |  |
| Orthodox | 60(6.27) | 221(10.08) | 248(11.83) | 5.56 | (5.09, 6.03) |
| Catholic | 1(2.22) | 1(3.20) | 3(10.03) | 7.81 | (7.48, 8.14) |
| Protestant | 16(4.57) | 74(6.63) | 94(8.24) | 3.67 | (3.34, 4.00) |
| Muslim | 36(6.31) | 103(8.06) | 130(9.21) | 2.90 | (2.49, 3.31) |
| Other | 6(12.58) | 2(4.59) | 7(10.23) | -2.35 | (-3.03, -1.67) |
| **Contraceptive use** |  |  |  |  |  |
| No method | 86(5.58) | 219(7.26) | 254(9.37) | 3.79 | (3.40, 4.18) |
| Folkloric method |  |  |  |  |  |
| Traditional method | 2(11.70) | 11(19.91) | 10(32.18) | 20.48 | (19.29, 21.67) |
| Modern method | 31(7.89) | 174(10.52) | 219(10.88) | 2.99 | (2.49, 3.49) |
| **Marital status** |  |  |  |  |  |
| In a Union | 100(6.00) | 342(8.43) | 398(9.54) | 3.54 | (3.13, 3.95) |
| Not in a Union | 18(6.68) | 62(9.19) | 77(14.36) | 7.68 | (7.13, 8.23) |
| **Women work status** |  |  |  |  |  |
| No | 72(5.31) | 224(7.87) | 227(7.44) | 2.13 | (1.80, 2.46) |
| Yes | 47(7.84) | 179(9.56) | 255(15.04) | 7.20 | (6.59, 7.81) |
| **Wealth status** |  |  |  |  |  |
| Poorest or poorer | 47(6.23) | 54(3.02) | 69(3.87) | -2.36 | (-2.67, -2.05) |
| Middle | 19(7.27) | 24(2.65) | 29(3.11) | -4.16 | (-4.51, -3.81) |
| Richer or richest | 53(5.65) | 324(16.25) | 385(18.82) | 13.17 | (12.51, 13.83) |
| **Read newspaper/ megazine** |  |  |  |  |  |
| No | 112(5.84) | 364(7.92) | 436(9.37) | 3.53 | (3.13, 3.93) |
| Yes | 6(33.35) | 39(30.64) | 46(48.30) | 14.95 | (12.67, 17.23) |
| **Listen radio** |  |  |  |  |  |
| No | 81(4.83) | 285(7.54) | 324(7.97) | 3.14 | (2.81, 3.47) |
| Yes | 38(13.76) | 119(12.55) | 158(23.29) | 9.53 | (8.52, 10.54) |

Table S1 (cont’d). Trends in the prevalence of overweight and obesity among women of reproductive age by characteristics (weighted) in 2005, 2011, and 2016

| **Characteristics** | **EDHS 2005 n(%)** | **EDHS 2011 n(%)** | **EDHS 2016 n(%)** | **Percentage point (%p) difference (2005-2016) 95%CI** | |
| --- | --- | --- | --- | --- | --- |
| **Listen/watch TV** |  |  |  |  |  |
| No | 84(4.53) | 240(5.86) | 246(5.94) | 1.41 | (1.14, 1.68) |
| Yes | 35(35.20) | 163(26.20) | 236(39.00) | 3.80 | (1.65, 5.95) |
| **Parity** |  |  |  |  |  |
| No child | 6(15.14) | 8(17.23) | 8(25.35) | 10.21 | (9.11, 11.31) |
| 1 to 3 children | 58(6.24) | 236(9.64) | 302(12.52) | 6.28 | (5.79, 6.77) |
| 4 and above children | 54(5.57) | 159(7.15) | 172(7.47) | 1.90 | (1.56, 2.24) |
| **Partner's education** |  |  |  |  |  |
| No education | 41(3.66) | 104(4.56) | 107(5.46) | 1.80 | (1.57, 2.03) |
| Primary | 23(4.58) | 148(8.03) | 106(6.84) | 2.26 | (1.97, 2.55) |
| Secondary | 38(16.08) | 67(21.54) | 79(20.77) | 4.69 | (3.66, 5.72) |
| Higher | 15(28.99) | 84(30.01) | 104(39.07) | 10.08 | (8.16, 12.00) |
| **Region** |  |  |  |  |  |
| Tigray | 1(0.70) | 20(7.07) | 29(9.30) | 8.60 | (8.31, 8.89) |
| Afar | 1(6.96) | 2(5.94) | 4(15.03) | 8.07 | (7.49, 8.65) |
| Amhara | 18(3.20) | 77(5.87) | 53(4.26) | 1.06 | (0.88, 1.24) |
| Oromia | 46(6.67) | 125(7.29) | 178(10.76) | 4.09 | (3.63, 4.55) |
| Somali | 12(17.73) | 24(28.46) | 29(23.56) | 5.83 | (4.68, 6.98) |
| Ben-Gumz | 0(2.78) | 2(3.76) | 4(7.64) | 4.86 | (4.60, 5.12) |
| SNNPR | 14(3.47) | 75(7.46) | 64(5.96) | 2.49 | (2.26, 2.72) |
| Gambela | 0(4.82) | 2(18.81) | 2(13.34) | 8.52 | (8.04, 9.00) |
| Harari | 1(15.79) | 2(28.68) | 3(31.02) | 15.23 | (13.96, 16.50) |
| Addis Ababa | 23(31.14) | 68(34.13) | 108(50.27) | 19.13 | (16.88, 21.38) |
| Dire Dawa | 2(22.06) | 5(40.24) | 9(34.19) | 12.13 | (10.58, 13.68) |
| **Place of residence** |  |  |  |  |  |
| Urban | 66(23.55) | 264(26.83) | 298(32.56) | 9.01 | (7.44, 10.58) |
| Rural | 53(3.15) | 139(3.72) | 184(4.81) | 1.66 | (1.47, 1.85) |
| **Regional-level education** |  |  |  |  |  |
| Low | 50(3.25) | 103(3.61) | 122(4.49) | 1.24 | (1.05, 1.43) |
| High | 69(16.33) | 300(16.15) | 360(17.74) | 1.41 | (0.43, 2.39) |
| **Regional-level wealth** |  |  |  |  |  |
| Low | 60(5.71) | 90(3.01) | 115(4.20) | -1.51 | (-1.80, -1.22) |
| High | 57(6.38) | 313(18.04) | 365(18.39) | 12.01 | (11.35, 12.67) |

Note: No education: those who did not attend school, Primary: those who completed grades 1-8, Secondary those who completed grades 9-12, and higher: those with a college certificate, diploma, or above (is based on EDHS category).
